# Is there an association between IFN-γ +874A/T polymorphism and periodontitis susceptibility? A meta-analysis

**DOI:** 10.1097/MD.0000000000007288

**Published:** 2017-06-23

**Authors:** Quan Shi, Chuan Cai, Juan Xu, Jinglong Liu, Hongchen Liu, Na Huo

**Affiliations:** Institute of Stomatology, Chinese PLA General Hospital, Beijing, China.

**Keywords:** gene polymorphism, interferon gamma, meta-analysis, periodontitis

## Abstract

**Background:**

Interferon-γ (IFN-γ) is a key proinflammatory cytokine which plays a critical role in the pathogenesis and progression of periodontitis. The single nucleotide polymorphism of +874A/T in human IFN-γ gene can influence the secretion of IFN-γ and affect periodontitis susceptibility. However, the findings of published studies are inconsistent. Therefore, the goal of this meta-analysis is to investigate whether there is an association between IFN-γ +874A/T polymorphism and periodontitis susceptibility.

**Methods:**

*PubMed* and the *Cochrane Library* were searched for eligible clinical studies. The odds ratio (OR) and 95% confidence interval (CI) of each study were pooled to estimate the strength of association between +874A/T and periodontitis. Subgroup analyses were performed to explore whether particular characteristics of studies were related to the overall results.

**Results:**

Seven studies and a total of 1252 periodontitis patients and 1622 periodontitis-free control subjects were included. No difference was observed in genotype distribution and allele frequency between periodontitis patients and control (T vs A: OR  =  1.01, 95% CI: 0.90–1.13, *P*  =  .878; TT vs AA: OR  =  1.07, 95% CI: 0.87–1.32, *P*  =  .537; AT vs AA: OR  =  1.00, 95% CI: 0.81–1.23, *P*  =  .996; TT+AT vs AA: OR  =  1.00, 95% CI: 0.84–1.19, *P*  =  .990; TT vs AA+AT: OR  =  1.03, 95% CI: 0.86–1.23, *P*  =  .733). Besides, the subgroup analysis based on ethnicity, type of periodontitis, and smoking status failed to identify significant differences in each model, either.

**Conclusions:**

The results of this meta-analysis suggest that IFN-γ +874 A/T polymorphism may not contribute to periodontitis susceptibility. High quality and well-designed studies which combine genetic and other environmental risk factors are needed to validate this conclusion in the future.

## Abstract 2

Supplemental Digital Content is available in the text

## 1 Introduction

As the world's second most common dental disease after dental decay, periodontitis is a slowly progressive form of destructive periodontal disease affecting 10% to 15% of the adult population and is the major cause of tooth loss in adults.^[1–4]^ In the American adult population, nearly half of those aged >30 have some level of periodontitis and almost 10% have severe conditions.^[5–7]^ Clinically, periodontitis is a chronic inflammatory disease that damages the soft and hard tissue supporting the structure of the teeth. If left untreated, periodontitis would result in loss of connective tissue attachment, erosion of alveolar bone, and loss of tooth eventually.^[8]^ Although periodontitis is initiated and sustained by periodontal pathogens, host inflammatory immune reaction, genetic and environmental factors also play a critical role in the pathogenesis and rate of progression of the disease.^[9–11]^

Many cytokines are involved in the process of periodontitis, including interleukins (ILs), tumor necrosis factor α (TNF-α), and interferon γ (IFN-γ). Interferons are a large family of cytokines that act against pathogens and tumors, and act as immunomodulatory factors.^[12,13]^ As the only member of the type II class of interferons, IFN-γ is one of the most critical mediators of immunity and inflammation, which is mainly secreted by CD4+Th cells, CD8+T cytotoxic cells, and natural killer cells.^[14,15]^ Accumulated evidence has supported that altered concentration of IFN-γ in gingival crevicular fluid, periodontal tissues, and serum is able to affect gingivitis, probing depth and alveolar bone loss.^[16–20]^ Moreover, increased serum levels of IFN-γ in periodontitis patients were shown to be associated with the enhanced dental plaque load with periodontal pathogens.^[21]^

Single nucleotide polymorphisms (SNPs) in genes may influence gene expression, protein function, and disease susceptibility. Reports have indicated that SNPs of the related inflammatory mediators may play a significant role in the risk of periodontal diseases by changing the protein expression and/or altering the immune response.^[22–24]^ As for the IFN-γ, there is an SNP +874A/T (rs2430561) located at the 5’-end of a CA repeat in the first intron of the human IFN-γ gene, which is related to the altered expression of IFN-γ.^[25,26]^ Some studies^[27,28]^ have analyzed the association between IFN-γ +874A/T polymorphism and the susceptibility or the severity of periodontitis, but the conclusions of these studies are inconsistent. Moreover, some studies have found that +874A/T polymorphism is also related to the occurrence of several periodontal pathogens (such as Aggregatibacter actinomycetemcomitans and Prevotella intermedia).^[29]^

Because of the inconclusive results of the available studies, the specific effect of IFN-γ +874A/T polymorphism on the susceptibility to periodontitis is still unclear. Therefore, we performed this meta-analysis to investigate whether there is an association between IFN-γ +874A/T polymorphism and periodontitis susceptibility, by which we hope to provide more evidence for understanding the pathogenesis and progression of periodontitis.

## 2 Methods

### 2.1 Literature search

We conducted a comprehensive literature search in *PubMed* and *Cochrane Library* database on September 26, 2016. The combination of the following key words and Mesh terms was used: periodontitis, periodontal disease, interferon gamma, IFN-gamma, and IFN-γ. The language of the published articles was restricted to English. Moreover, references in the related studies or reviews were also reviewed by manual searching to identify other potentially eligible studies. Ethical approval and informed consent were not required as this study was based on previously published studies and had no direct patient contact or influences on patient care.

### 2.2 Inclusion and exclusion criteria

In this meta-analysis, the following criteria were designed and used for including the identified studies: clinical studies focused on the association between IFN-γ +874A/T gene polymorphism and periodontitis risk; the frequencies of alleles or genotypes in case (periodontitis patients) and control (periodontitis-free subjects) groups can be extracted; periodontal patients and control subjects are clearly described and confirmed; studies use validated genotyping methods. The exclusion criteria were animal studies or in vitro studies; reviews, letters, case reports or comments; studies without available data that could be extracted.

Based on the above criteria, the search results were independently assessed by 2 reviewers (SQ and CC), and any disagreement was resolved through discussion with a third reviewer (LHC).

### 2.3 Data extraction

The data extraction was performed under a predefined form by 2 reviewers (SQ and CC) independently. Disputes were settled by discussion with a third reviewer (LHC). The following information was extracted from each included study: first author, year of publication, country, characteristics of the subjects (including the number of patients in both groups, age, and sex, smoking status), ethnicity (Asians, Caucasians, and others), genotyping method, alleles or genotypes frequency in cases and controls.

### 2.4 Quality assessment and Hardy–Weinberg equilibrium

Methodological quality was evaluated by 2 researchers (XJ and LJL) according to a methodological quality assessment scale adopted from previous publications.^[30,31]^ According to this scale, representativeness of cases, source of controls, sample size, quality control of genotyping methods, and Hardy–Weinberg equilibrium (HWE) were used to appraise the methodological quality of the included studies with a maximum of 10 points (Supplementary Table S1). The scores of 0 to 4, 5 to 7, and 8 o 10 indicated poor, fair, and good study quality, respectively.

As for the HWE, it was evaluated for each study by χ^2^ test in control groups based on the genotyping distribution in control subjects, and *P* < .05 was considered a significant departure from HWE.

### 2.5 Statistical analyses

Statistical analyses were performed by using STATA software (Version 12.0; Stata Corp, College Station, TX). The odds ratio (OR) value and the 95% confidence interval (CI) of each study were pooled to estimate the strength of association between IFN-γ +874A/T polymorphism and periodontitis susceptibility. Pooled ORs were calculated for allelic model (T vs A), homozygote model (TT vs AA), heterozygote model (AT vs AA), dominant model (TT+AT vs AA), and recessive model (TT vs AA+AT). The statistical heterogeneity was verified by *I*^2^ statistics. Fixed-effect model was adopted to estimate the OR and 95% CI when heterogeneity was low (*I*^2^ < 50%), while the random effects was used when heterogeneity was high (*I*^2^ > 50%). Sensitivity analysis was performed to analyze the stability of the pooled results. Publication bias in each model was detected by Egger test.

Besides, subgroup analyses were performed to explore whether particular characteristics of studies (ethnicity, type of periodontitis, results of HWE, and smoking status) were related to the value of the overall OR and 95% CI. All the *P* values were 2-sided and *P* < .05 was considered statistically significant.

## 3 Results

### 3.1 Characteristics of included studies

**Figure 1:**
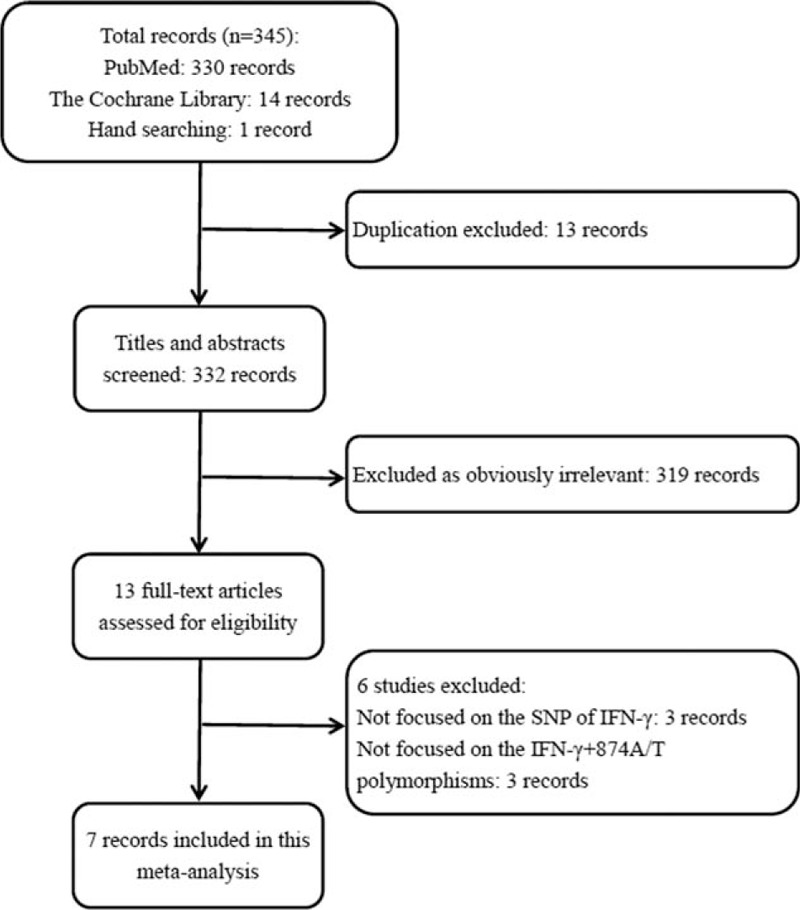
Flow diagram for selection of studies. SNP  =  single nucleotide polymorphism.

A flow chart of the study selection process is shown in Figure 1. A total of 345 published studies were identified from different databases and by hand searching. After the exclusion of the duplicated records, 332 studies were left for screening. Then through reading the titles and abstracts, 319 of the 332 articles not related to our focused topic were excluded, leaving 13 articles for further full-text review. Eventually, 7 studies^[27–29,32–35]^ were included in this meta-analysis according to the inclusion criteria.

The publication dates of the 7 included studies ranged from 2006 to 2015, in which a total of 1252 periodontitis patients and 1622 periodontitis-free control subjects from 6 different countries were studied. Two of included studies reported on the Asians,^[27,32]^ while the other 5 studies reported on the Caucasians.^[28,29,33–35]^ Besides, in terms of the type of periodontitis, 5 studies focused on chronic periodontitis (CP),^[27,28,32,33,35]^ one study focused on aggressive periodontitis (AgP),^[34]^ and the last one focused on both CP and AgP.^[29]^ The objects of 3 studies were non-smokers, while the other 3 studies were including both smokers and non-smokers (mixed group), and the last one did not give this information.^[32]^ The characteristics of the included studies and patients are shown in Table 1.

**Table 1:**
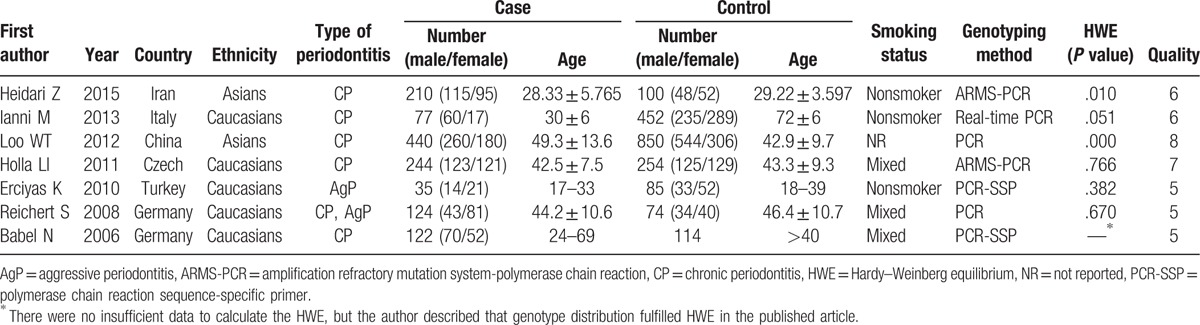
Characteristics of studies included in the meta-analysis.

Among the 7 included studies, the genotype distributions in the control subjects of 4 studies were in accordance with HWE,^[28,29,33,34]^ while 2 studies deprived from HWE.^[27,32]^ In the study of Babel et al,^[35]^ insufficient data made it impossible to calculate the HWE, but the authors described that genotype distribution fulfilled HWE in the published article. All included studies had a quality score ≥5 (moderate–high quality, Table 1 and Supplementary Table S2).

### 3.2 Meta-analysis results

#### 3.2.1 Overall OR and 95% CI

**Figure 2:**
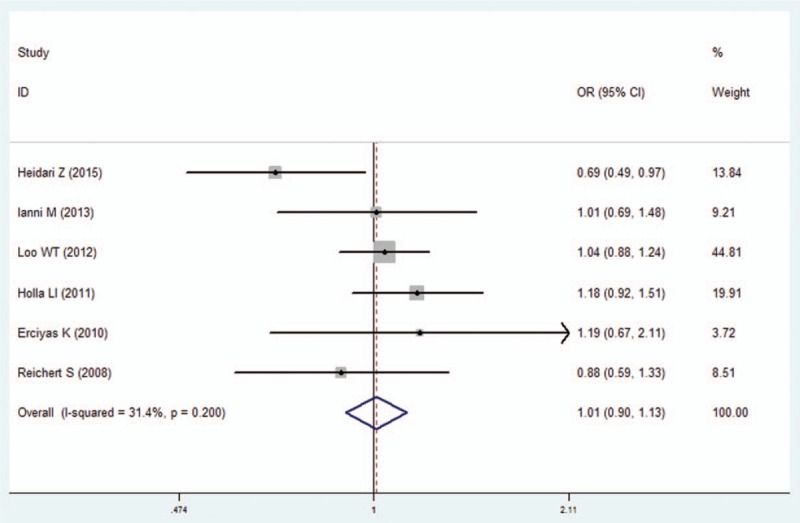
Forest plot of the IFN-γ +874A/T polymorphism and periodontitis susceptibility in allelic model (T vs A). CI  =  confidence interval, OR  =  odds ratio.

**Figure 3:**
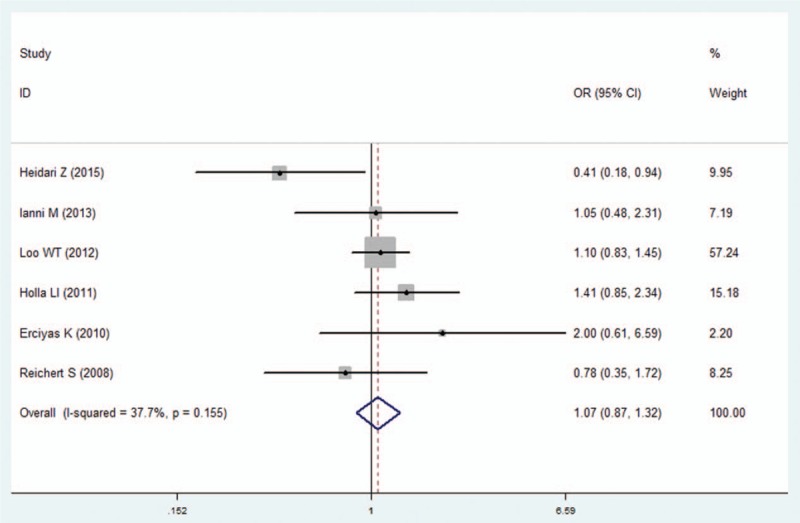
Forest plot of the IFN-γ +874A/T polymorphism and periodontitis susceptibility in homozygote model (TT vs AA). CI  =  confidence interval, OR  =  odds ratio.

**Figure 4:**
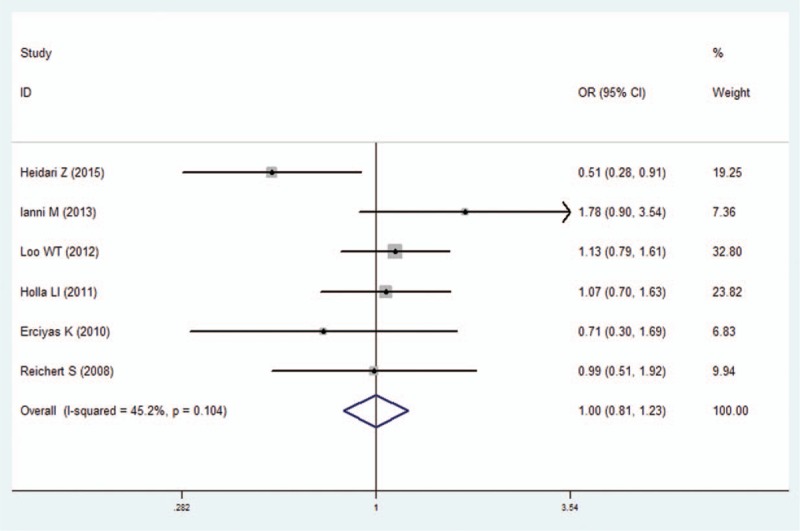
Forest plot of the IFN-γ +874A/T polymorphism and periodontitis susceptibility in heterozygote model (AT vs AA). CI  =  confidence interval, OR  =  odds ratio.

**Figure 5:**
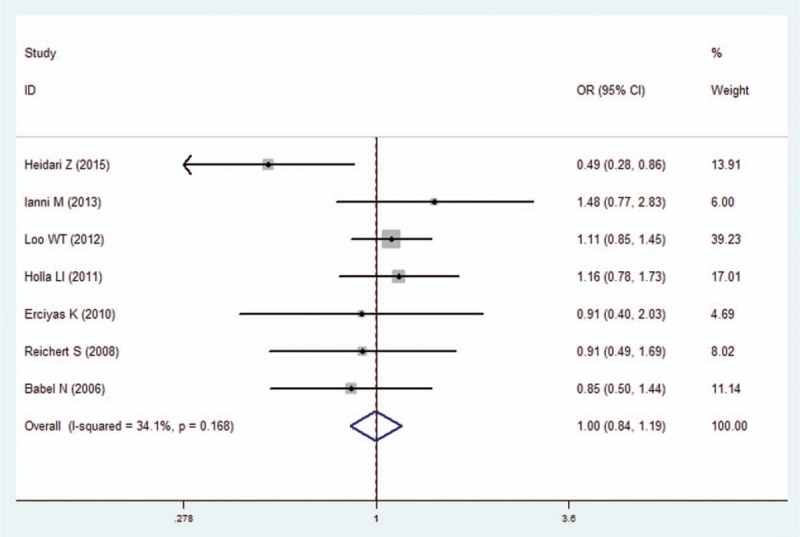
Forest plot of the IFN-γ +874A/T polymorphism and periodontitis susceptibility in dominant model (TT+AT vs AA). CI  =  confidence interval, OR  =  odds ratio.

**Figure 6:**
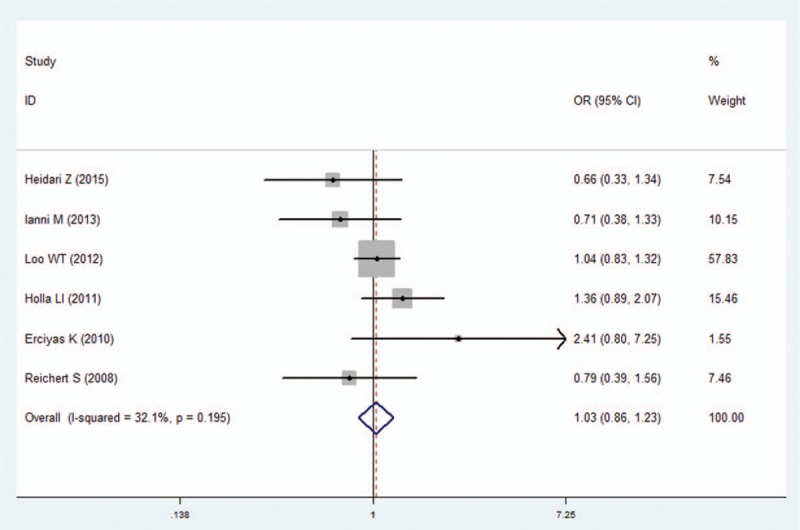
Forest plot of the IFN-γ +874A/T polymorphism and periodontitis susceptibility in recessive model (TT vs AA+AT). CI  =  confidence interval, OR  =  odds ratio.

We pooled all of the included studies to estimate the association between IFN-γ +874A/T polymorphism and periodontitis. No significant heterogeneity was identified by *I*^2^ statistic in all of the genetic models (Table 2), therefore fixed-effects model was used in this analysis. No difference was observed in genotype distribution and allele frequency between periodontitis patients and control, which means no significant association was identified between IFN-γ +874A/T polymorphism and periodontitis by the comparison of 5 genetic models (T vs A: OR  =  1.01, 95% CI: 0.90–1.13, *P*  =  .878; TT vs AA: OR  =  1.07, 95% CI: 0.87–1.32, *P*  =  .537; AT vs AA: OR  =  1.00, 95% CI: 0.81–1.23, *P*  =  .996; TT+AT vs AA: OR  =  1.00, 95% CI: 0.84–1.19, *P*  =  .990; TT vs AA+AT: OR  =  1.03, 95% CI: 0.86–1.23, *P*  =  .733; Figs. 2–6, Table 2).

**Table 2:**
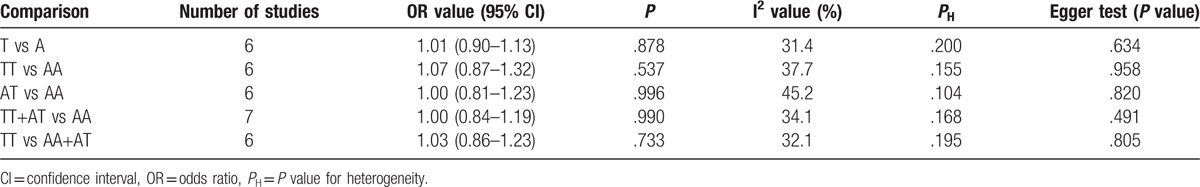
Summary of the association between IFN-γ +874 polymorphisms and periodontitis.

In the sensitivity analysis, the influence of each individual study on the pooled OR was assessed by removing one study each time in each genetic model. The results revealed that the overall ORs did not significantly differ (Supplementary Figure S1), suggesting the stability of the results in this meta-analysis. Furthermore, by Egger regression test, no significant publication bias was identified in overall comparisons (Table 2).

#### 3.2.2 Subgroup analysis

To evaluate the possible effect of ethnicity, type of periodontitis, results of HWE, and smoking status on the variability of overall estimated OR values, we performed 2 subgroup analyses. First, the included studies were divided into Asians and Caucasians, but no association was found in all of the comparison models in both of these 2 subgroups (Table 3). Both of the studies reported on Asians^[27,32]^ were deprived from HWE, therefore the results of subgroup based on the HWE were the same as those of the ethic subgroup. Both of these subgroup analyses disclosed a significant association between IFN-γ +874A/T polymorphism and periodontitis susceptibility.

**Table 3:**
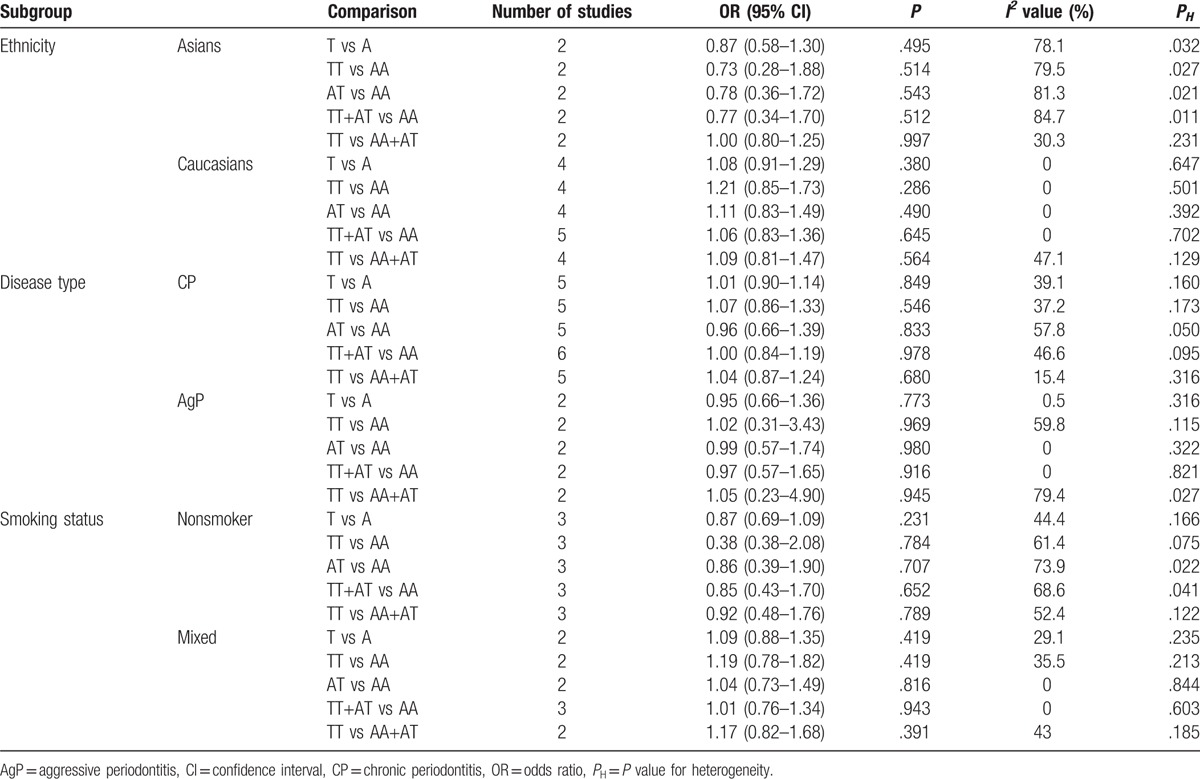
Statistics for subgroup analysis.

Further subgroup analysis was conducted based on the type of periodontitis and the studies were divided into CP and AgP subgroups. However, the results showed that there were no statistical differences in each model both in the CP and AgP subgroups, either (Table 3). At last, the subgroup analysis based on smoking status, while the results showed no significant association between IFN-γ +874A/T polymorphism and periodontitis susceptibility in both the nonsmoker group and mixed group (Table 3).

## 4 Discussion

Periodontitis is regarded as a chronic inflammatory condition, which is caused by specific pathogenic bacteria and influenced by environmental and genetic factors.^[36,37]^ Genetic variants of inflammatory cytokines have been confirmed to be associated with susceptibility to periodontitis.^[35,38]^ For example, many clinical studies and meta-analysis have found that polymorphisms of IL-1α, IL-1β, IL-4, and TNF-α may contribute to the susceptibility of periodontitis.^[38–41]^ As a regulatory key in immune response and inflammation process, IFN-γ has been confirmed to be present at high levels in diseased periodontal tissues, and to be associated with progressive lesions and severity of periodontal diseases.^[16,42]^ The present meta-analysis was conducted to evaluate the association between IFN-γ +874A/T polymorphism and periodontitis susceptibility.

The SNP of some genes could cause a change in the protein expression. As for +874A/T, there are 3 possible genotypes: the AA, AT, and TT genotypes, which are thought to represent 3 different expression levels of IFN-γ: low, intermediate, and high, respectively.^[26,33]^ The different level of IFN-γ can affect the immune response and susceptibility to inflammatory diseases.^[43,44]^ Reichert et al^[29]^ concluded that IFN-γ TT genotype could have a lower susceptibility to periodontitis because of the high quantity of cytokine production in this genotype. Moreover, Heidari et al^[27]^ found that IFN-γ +874A/T genetic polymorphism was linked with susceptibility to periodontitis in Iranian population, and the T allele was less frequent in the periodontitis compared with control subjects.^[27]^ However, in this meta-analysis, the TT genotype distribution and T allele in the periodontitis patients had no difference compared with the control subjects (TT vs AA: OR  =  1.07, 95% CI: 0.87–1.32, *P*  =  .537; T vs A: OR  =  1.01, 95% CI: 0.90–1.13, *P* =.878; Table 2). Besides, we uncovered a positive relationship between +874A/T polymorphism and periodontitis susceptibility in other comparison models. To analyze the stability of the pooled results, sensitivity analysis by omitting each included studies was conducted in this meta-analysis. The results revealed that the overall ORs were not significantly different. Furthermore, the Egger regression test revealed that no significant publication bias was identified.

The inconsistency of the results of +874A/T polymorphism and periodontitis risk may be attributed to several factors. First, the relationship between SNPs and diseases may be influenced by the race. In this meta-analysis, included studies involve patients with different racial and ethnic backgrounds. Second, periodontitis can be divided into several types, while the current studies mainly focus on the CP and AgP. Third, the design of the studies may also contribute to the inconsistency. For example, as the periodontitis is a multifactorial disease, the environmental factors may play a significant role in the risk of periodontal diseases, but the control subjects in each included studies matched different kind of the confounding factors and revealed different HWE results. Fourth, smoking is one of the risk factors which are responsible for periodontitis.^[45]^ Of the seven included studies, the objects of 3 studies were nonsmokers, 3 were including smokers and the last one did not give this information. Furthermore, as a multiple factor disease, age and sex may also play a critical role in the pathogenesis and development of periodontitis. Considering the above factors, despite that the overall heterogeneity was low in this study, we performed subgroup analysis to explore the association between IFN-γ +874A/T polymorphism and periodontitis susceptibility in different ethnicity, periodontitis type, and HWE results.

In this meta-analysis, 5 studies^[28,29,33–35]^ reported on the Caucasians and 2 studies^[27,32]^ focused on the Asians. In the subgroup analysis, no association was found in all of the comparison models in Asians and Caucasians. However, this finding should be interpreted with caution in the Asians subgroup. The *I*^2^ statistic revealed that in the Asians subgroup, there was a trend of increasing heterogeneity, compared with overall test and Caucasians subgroup. This may be caused by the limited study number and sample size. In addition, genotypes from the 2 studies^[27,32]^ in Asians subgroup were deprived from HWE, which may contribute to the heterogeneity and affect the reliability of the results. Therefore, larger sample, well-designed studies are needed in the future.

CP and AgP are both regarded as destructive periodontal diseases,^[46]^ but they are different in terms of the severity and rate of periodontal attachment loss and bone destruction. To evaluate the possible effect of type of periodontitis on the variability of overall estimated OR values, we divided the included studies into CP and AgP subgroup. Five studies^[27,28,32,33,35]^ focused on the CP, one^[34]^ focused on the AgP and the last one^[29]^ focused on both. The comprehensive analysis failed to identify a significant association between IFN-γ +874A/T polymorphism and periodontitis susceptibility in both CP and AgP subgroups.

Smoking is an important risk factor which can increases the occurrence of periodontitis, directly or indirectly.^[45,47]^ Moreover, the smoking status may affect the results. Therefore, to investigate whether the smoking status could affect the association between IFN-γ +874A/T polymorphism and periodontitis susceptibility, we divided the studies into nonsmoker subgroup and mixed group. As with all the other subgroup analyses, no significant association between IFN-γ +874A/T polymorphism and periodontitis susceptibility in both the nonsmoker group and mixed group. Our results are similar to the study of Ianni et al^[28]^ and Erciyas et al^[34]^ However, opposite results were found in the study of Heidari et al,^[27]^ in which significant difference was found in genotype and allele frequency of IFN-γ +874A/T gene polymorphism in nonsmoker patients and nonsmoker controls. As described above, some factors, including the design of the studies and race, may contribute to these different conclusions.

Although in our meta-analysis no significant association was identified between IFN-γ +874A/T polymorphism and periodontitis, accumulated evidence has revealed that this SNP is associated with many diseases.^[48–53]^ A meta-analysis has indicated that IFN-γ +874A/T polymorphism is associated with increased genetic susceptibility to autoimmune diseases, especially in idiopathic thrombocytopenic purpura and systemic lupus erythematosus (SLE).^[48]^ Besides, IFN-γ +874A/T polymorphism may increase the risk of recurrent pregnancy loss in the non-Caucasians.^[51]^ Moreover, it is still controversial about whether Interferon-γ +874A/T polymorphism could increase the cancer risk. Through a 9 study-based meta-analysis, Sun et al^[49]^ found that IFN-γ +874A/T polymorphism was likely to increase the risk of cervical cancer, while Ge et al^[54]^ concluded that IFN-γ +874A/T polymorphism may not contribute to cancer susceptibility. Hence, well-designed and large-scale studies are demanded.

To our knowledge, this is the first meta-analysis to estimate the association between IFN-γ +874A/T polymorphism and periodontitis risk by quantitative analysis. We conducted both database and hand searching to identify the potentially eligible studies as completely as possible. The methodological quality assessment results revealed that the included studies had moderate–high quality. Furthermore, sensitivity analysis and subgroup analysis were performed to analyze the stability of the pooled results in this meta-analysis. Nevertheless, there are still some weaknesses in this meta-analysis. First, the studies are confined to English-language studies, which might have language bias. Second, the heterogeneity between studies was reflected in some comparison models, which may affect stability, even if we used random effect model, which may affect the stability of the results. Third, because of limited information provided by the included studies, we did not explore some factors, such as age and sex, could affect the overall effect. Fourth, the number of eligible studies and patients was limited in some subgroups, therefore, more high quality related studies are needed to further explore the association between IFN-γ +874A/T polymorphism and periodontitis susceptibility.

## 5 Conclusions

In summary, the results of our meta-analysis failed to find a significant association between IFN-γ +874A/T polymorphism and periodontitis susceptibility based on current evidence. Considering the limitations in this meta-analysis and the available clinical studies, high-quality and well-designed studies which combine genetic and other environmental risk factors are needed to validate the conclusion in the present study in the future, especially for the Asian population.

## Supplementary Material

Supplemental Digital Content
