# Supplementary material for: Is there an association between IFN-γ +874A/T polymorphism and periodontitis susceptibility? A meta-analysis
Source: Medicine (Baltimore). 2017 Jun 23;96(25):e7288. doi: 10.1097/MD.0000000000007288 (PMC5484252; doi:10.1097/MD.0000000000007288)
Supplement: Supplemental Digital Content [file medi-96-e7288-s001.doc]

Legends to Supplementary figures:

**Figure S1: Sensitivity analysis of the meta-analysis. A: comparison in allelic model (T vs A). B: comparison in homozygote model (TT vs AA). C: comparison in heterozygote model (AT vs AA). D: comparison in allelic model dominant model (TT+AT vs AA). E: comparison in recessive model (TT vs AA+AT).**


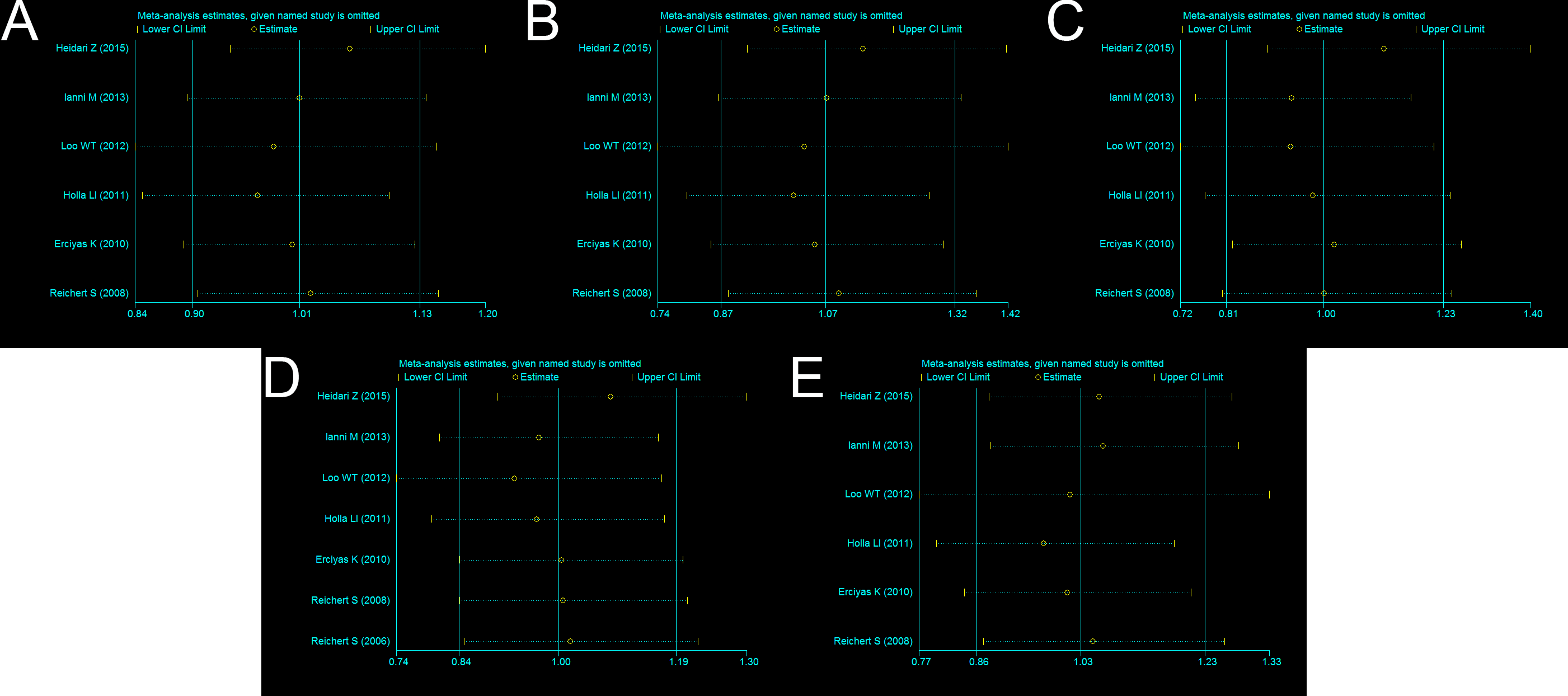


| **Table S1. Scale for methodological quality assessment.** | |
| --- | --- |
| Criteria | Score |
| 1.Representativeness of cases |  |
| Periodontitis diagnosed according to acknowledged criteria. | 2 |
| Mentioned the diagnosed criteria but not specifically described. | 1 |
| Not Mentioned. | 0 |
| 2.Source of controls |  |
| Population or community based | 3 |
| Hospital-based periodontitis-free controls | 2 |
| Healthy volunteers without total description | 1 |
| Periodontitis-free controls with related diseases | 0.5 |
| Not described | 0 |
| 3.Sample size |  |
| >200 | 2 |
| 80-200 | 1 |
| <80 | 0 |
| 4.Quality control of genotyping methods |  |
| Repetition of partial/total tested samples with a different method | 2 |
| Repetition of partial/total tested samples with the same method | 1 |
| Not described | 0 |
| 5.Hardy-Weinberg equilibrium (HWE) |  |
| Hardy-Weinberg equilibrium in control subjects | 1 |
| Hardy-Weinberg disequilibrium in control subjects | 0 |

| First author | Year | Representativeness of cases | Source of controls | Sample size | Quality control of genotyping methods | HWE | Total Score of quality assessment scale |
| --- | --- | --- | --- | --- | --- | --- | --- |
|
| Heidari Z | 2015 | 2 | 2 | 2 | 0 | 0 | 6 |
| Ianni M | 2013 | 2 | 1 | 2 | 0 | 1 | 6 |
| Loo WT | 2012 | 2 | 3 | 2 | 1 | 0 | 8 |
| Holla LI | 2011 | 2 | 2 | 2 | 0 | 1 | 7 |
| Erciyas K | 2010 | 2 | 1 | 1 | 0 | 1 | 5 |
| Reichert S | 2008 | 2 | 1 | 1 | 0 | 1 | 5 |
| Babel N | 2006 | 2 | 1 | 2 | 0 | 0 | 5 |

**Table S2. Methodological quality of the included studies**

HWE：Hardy–Weinberg equilibrium
